# Biobanking and risk assessment: a comprehensive typology of risks for an adaptive risk governance

**DOI:** 10.1186/s40504-021-00117-7

**Published:** 2021-12-13

**Authors:** Kaya Akyüz, Gauthier Chassang, Melanie Goisauf, Łukasz Kozera, Signe Mezinska, Olga Tzortzatou, Michaela Th. Mayrhofer

**Affiliations:** 1 BBMRI-ERIC, Graz, Austria; 2 Department of Science and Technology Studies, University of Vienna, Vienna, Austria; 3 CERPOP, Université de Toulouse, Inserm, Université Paul Sabatier, Toulouse, France; 4 Institute of Clinical and Preventive Medicine, University of Latvia, Riga, Latvia; 5 Biomedical Research Foundation of the Academy of Athens, Athens, Greece

**Keywords:** Biobanking, Biobank management, Risk governance, Risk assessment, ELSI, Data privacy, Typology, Sustainability, Stakeholders

## Abstract

Biobanks act as the custodians for the access to and  responsible use of human biological samples and related data that have been generously donated by individuals to serve the public interest and scientific advances in the health research realm. Risk assessment has become a daily practice for biobanks and has been discussed from different perspectives. This paper aims to provide a literature review on risk assessment in order to put together a comprehensive typology of diverse risks biobanks could potentially face. Methodologically set as a typology, the conceptual approach used in this paper is based on the interdisciplinary analysis of scientific literature, the relevant ethical and legal instruments and practices in biobanking to identify how risks are assessed, considered and mitigated. Through an interdisciplinary mapping exercise, we have produced a typology of potential risks in biobanking, taking into consideration the perspectives of different stakeholders, such as institutional actors and publics, including participants and representative organizations. With this approach, we have identified the following risk types: economic, infrastructural, institutional, research community risks and participant’s risks. The paper concludes by highlighting the necessity of an adaptive risk governance as an integral part of good governance in biobanking. In this regard, it contributes to sustainability in biobanking by assisting in the design of relevant risk management practices, where they are not already in place or require an update. The typology is intended to be useful from the early stages of establishing such a complex and multileveled biomedical infrastructure as well as to provide a catalogue of risks for improving the risk management practices already in place.

## Introduction

Recent years have seen an expansion of existing biobanking structures and emergence of new biobanks focusing on populations, diseases, biological samples and data, leading to an above-average increase in research that make use of these infrastructures (Astrin and Betsou 2016) as well as expansion of biobanks into greater networks (Ortega-Paíno and Tupasela 2019). Rather than collecting samples and data for a specific project as in classical clinical research, the storage of samples and data for potential research in the future is a hallmark of biobanking (Mikkelsen et al. 2019). The risks that are associated with participation in clinical research had been central to the practice of informed consent and research ethics; however, with the expansion of biobanking infrastructures, a more comprehensive understanding, communication and mitigation of risks has become a necessity along with the importance of developing useful tools in order to evaluate and manage them, and is already part of successful, sustainable biobanks.

Despite major transformations in the infrastructures for biomedical research, however, the assessment, management and communication of risks, in other words, risk governance (Jacobson, McHugh, and Tran 2013) in biobanking relies mainly on adopting the international guidelines that aim for standardization, such as quality management: International Organization for Standardization (ISO) 20387: 2018 Biotechnology – Biobanking – General requirements for biobanking (ISO 2018a). Considering ISO beyond biobanking, risk is a topic with its own standards of risk management (ISO 2018b) and risk assessment techniques (ISO 2019). However, risk by definition involves uncertainty and efforts towards standardization notwithstanding, the contextuality of the risks calls for a more nuanced understanding, where the standardized methods of managing emerging risks often lag behind the identification of these risks. In this article, we provide a comprehensive catalogue of potential risks in biobanking, not only for the participant or various stakeholders, but also for the biobank, biobank employees, biobanking community or even broader arena of biomedical research. This typology of risks is not to be regarded as complete or rigid in structure, but rather a framework to approach risk management as an active process that constantly needs to be reviewed and updated. In this regard, this article presents a plethora of risks; the acknowledgment of such risks as a first step is then the beginning of a process that ensures that biobanks can identify, minimize or even prevent the risks relevant in their specific context and ultimately their realization in collaboration with contributing clinicians and researchers. Indeed, while the typology is starting out with biobanks in focus, many of the risks discussed are themselves part and parcel to life sciences research that involves humans. Therefore, conceiving efficient risk management practices in biobanking relies on capacities of co-construction and fluid collaboration between several stakeholders, including participants, all having their role in appropriate custodianship of the samples and data. Considering the experience of scientific institutions on risk mitigation and communication, biobanking is building on lessons learned from the past and benefiting from a long-term experience of academic research that has contributed to human health.

Changing temporality of the risks in research is one of the major drivers for the proposed conceptualization of risks. A major transformation in this regard is the switch from study-specific consent to broad consent as in the case of many biobanks, which adds a further layer into the communication and understanding of risks (Mikkelsen et al. 2019). In the study-specific consent model, the participant’s main risks are physical and often relevant only for the duration of providing the sample except for invasive studies as well as pharmaceutical research (Helgesson 2012). In broad consent models used in many biobanks, although the physical risks are often minimal, the non-physical risks may need a careful evaluation and balancing in a long-term perspective. As the individual cannot be entirely informed of the future risks, only some of which can be anticipated at the time of consent, the practice of consent relies on the assumption that emerging risks can be mitigated by the biobank in the future. Therefore, during the recruitment process, the participant is not only informed about risks, but ideally also assured of the expected scientific benefits from her/his participation, of the aim and scope, goals and values of the biobank and its governance model that can adapt to changing risks in light of societal, technological and scientific developments (Prainsack and Buyx 2013, Harmon 2009).

Context-dependence of the risks is a second aspect at the heart of this article. Identification of risk often relies on categorization of potential harm as sufficiently likely and sufficiently severe (Mikkelsen et al. 2019). However, severity or likeliness can change, for instance, in the case of genomic identifiability (Malin et al. 2011, Kasperbauer et al. 2018) due to proliferation of publicly available online genomic data, which has raised privacy concerns regarding research infrastructures, as well as recreational websites, and big data practices such as data mining (Gymrek et al. 2013, Erlich and Narayanan 2014, Erlich et al. 2018, Conboy 2020). At the same time, risks are interpreted very differently by individuals and these interpretations do not always align with the clinical or epidemiological risk rationalities (Lupton 2013, Quinn et al. 2013), nor are they always seen relevant by the individual in making their decision (Helgesson 2012). For instance, citizens’ preferences for consent models in biobanking can be shaped and contextualized by their concerns about the “appropriateness” of research practice and developments as manifested with uncertainty toward samples and data uses, a lack of knowledge about biobanking practices and risks, and an unclear future connected to unknown purposes of sample and data use (Goisauf and Durnová 2018). Risks may be seen as objective; however, they are perceived very subjectively and interpreted within individual’s past experiences and values (Lupton 2013, Quinn et al. 2013).

A simple taxonomic categorization may give the impression that the mentioned risks are easily separable; however, risks are often interrelated and thus necessitate a holistic thinking to manage them successfully. A controversy, such as the Havasupai case (Garrison 2012, Quinn et al. 2013, Tzortzatou 2015, Helgesson 2012), may entail numerous risks at the same time, from the violation of participants’ values to economic risks due to the legal action, rising rates of withdrawals to recruitment bias. Just like the exclusion of certain groups from participation may result in lower generalizability of findings (Prictor, Teare, and Kaye 2018), past controversies involving misconduct and not communicating and mitigating risks may lead to mistrust in biobanking as exemplified by those focusing on indigenous communities (Tauali`i et al. 2014), thus hampering the scientific and technological potential of the infrastructures. Against the background of interrelated risks, the social license that is necessary for a biobank to function relies on continuous societal support, and in this regard, a good governance with transparency, accountability and oversight has a positive impact and is strongly needed (Gille, Vayena, and Blasimme 2020, Gehman, Lefsrud, and Fast 2017, Parsons and Moffat 2014). With the proposed typology, we strive towards this goal with a plastic structure to assess and review risks in biobanking. A comprehensive typology of risks, in this regard, would allow better communication and mitigation of risks, as well as better coordination and engagement of the different stakeholders in biobanking process, thus contributing to biobanks’ capacity to support research and innovation, raising standards of responsibility and accountability.

## Materials and methods

The typology that this paper proposes, is methodologically based on a conceptual research design that aims to categorize, classify and organize distinct variants and types of a phenomenon (Jaakkola 2020). Such an approach provides both, a comprehensive as well as “precise and nuanced understanding of a phenomenon” and its “key dimensions” (p. 23). The methodology enables to structure a “fragmented research domain” which entails “differing manifestations of a concept”, in our case risks in biobanking (p. 24). Considering that risk as a concept in biobanking is used in various ways and in multiple situations, from everyday use to systematic and standardized risk assessment procedures, the aim of the present study is to produce a practice-oriented map of the identified risk types and their elements.

To gather different manifestations and uses of the ‘risk’ concept, the analysis builds on both scientific literature and policy documents to include theoretical concepts and practical applications relevant for biobanks (e.g., on legal aspects such as participants’ rights, personal data protection, or on technical aspects such as specific quality constraints ensuring scientific value of the activity). Following the approach of a typology outlined before (Jaakkola 2020), the sampling of the material was guided by the expertise of the co-authors – in ethics, law, biomedicine, political science, sociology, science and technology studies – with the goal to provide an overview and synthesis of the state of the art, to link approaches from various disciplines and to balance the perspectives on risks in the field of biobanking. In going beyond a mere descriptive summary of the literature, the proposed typology was generated through inductively identifying and capturing relevant characteristics and variants of the concept from the material. Finally, based on the experience in biobanking, the coauthors reflected on the application of the developed typology to different situations, where engagements with biobankers during workshops and presentations of the previous versions of the typology contributed to its further crystallization.

## Typology of risks

**Fig. 1:**
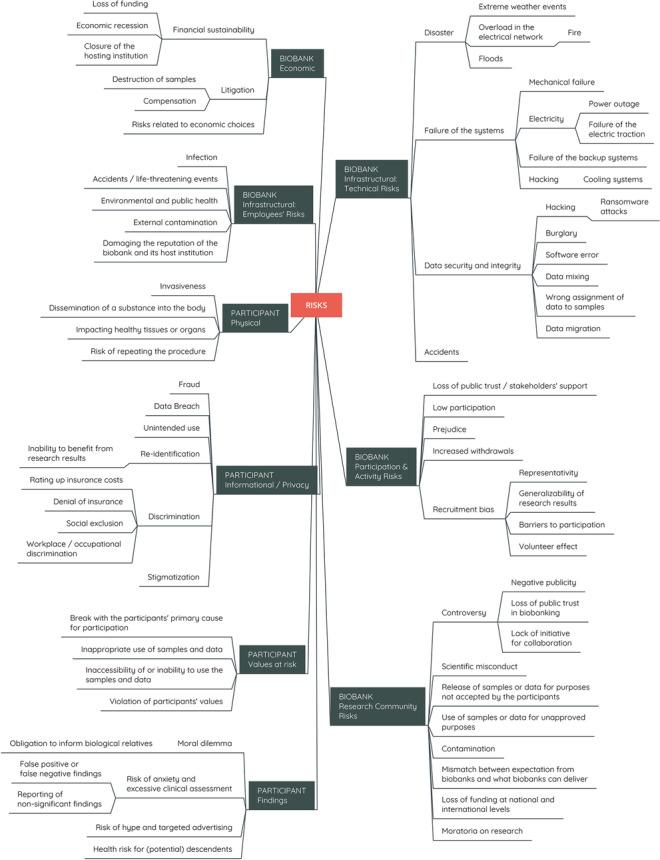
Typology of risks in biobanking. The discussed risks are visualized according to the identified types

A typology, by definition, may be limiting since one of its aims is to group distinct entities into generated types where the variations within types are temporarily dissolved. It may also be an impossible endeavor as the same entity may have a multiplicity that allows it to be categorized under different types or linked to other types. Despite efforts towards a balanced typology, certain types become more prominent, whereas others blend into the background. Finally, typologies are ‘constructed’ in that they necessitate reflections on the spatio-temporal aspects of their representational capacity. Thus, the typology of risks in biobanking (Fig. 1) cannot be considered a simple categorization exercise. It is rather an object to highlight the internal nuances, interrelatedness and multiplicity of types, emphasizing aspects that are sidelined in discussion of risks in biobanking while acknowledging that any typology of risks will be temporally and spatially situated.

In the following sections, risks in biobanking will be considered from two distinct perspectives. First, the focus will be on the biobank side, examining assessment and management of economic risks, infrastructural risks (technical and employees), risks related to participation and activity, and finally, research community risks. Then, the typology will focus on the participant, where physical risks, informational/privacy risks, values, and risks related to findings will be discussed.

### Biobank risks

Taking the biobank in the center, we have focused on five levels: the economic, infrastructural - technical and employees, biobanking practice (participation and activity) and research community. Recounting all potential risks would have limited the purpose of this article. Likewise, a complete separation of these levels from each other and the second major category of participant’s risks is not possible. Therefore, the descriptions of the types below are supplemented with complementary elements within the other types.

#### Economic risks of the biobank

Although the biobanking infrastructure is usually located within the organizational structure of a university or other scientific institution, the economic sustainability might become a bottleneck due to high maintenance costs (Sargsyan et al. 2015, Clément et al. 2014). Financial sustainability is one of the main issues that should be carefully analyzed before setting up a biobank, and as a part of the long-term operation plan (Gee et al. 2015). Research grants or seed funds end relatively quickly and there are situations in which they are not replaced by new projects. Therefore, some biobanking units consider alternative sources of funding that will guarantee financial independence and the ability to cover basic activities, such as the maintenance of staff, equipment servicing and utility bills, e.g. for electricity and gases, which are largely consumed by the freezing system (Henderson, Goldring, and Simeon-Dubach 2019). Opening the biobank to the external market by offering services to other institutions that do not have a biobank in their structure, generating data that are released instead of samples, and cooperation between public-private sectors are the main possibilities that could be considered when planning a sustainability model for a biobanking facility (Hofman et al. 2014, Henderson, Goldring, and Simeon-Dubach 2019). The choices made in order to minimize economic risks themselves carry certain risks. For instance, prioritization of data over samples or prioritization of certain data over other types of data (e.g., epigenomic data or single cell sequencing) due to costs may in the long run be a limiting factor for research as much as it is taking advantage of an opportunity to collect large amounts of data for data mining. For instance, many biobanks allow genome-wide association studies (GWAS) with large-scale genotyping of single nucleotide polymorphisms (SNPs) and such studies often lead to identification of variants that may be responsible for analyzed health conditions; at the same time they may be inherently limited since alternative data types (e.g., whole genome sequence or epigenomic data) and their contribution to studied health condition (e.g., contribution of rare variants or epigenetic regulation in comparison to common variants) may be more relevant (Tam et al. 2019).

Research economists together with biobankers have developed many business models focusing on biobank activities. The key to safeguard the functioning of the biobank is a comprehensive approach that goes beyond laboratory costs and focuses on all activities that affect the collection of human biological material, including clinical, information technologies and administrative services (Clément et al. 2014). The comprehensive business model also enables methodological activities to be performed in order to optimize costs, especially under financial constraints. On top of these costs, biobanks should also consider further costs related to communication and dissemination as well as direct engagements with participants, e.g. in case of incidental or secondary findings, their validation and return (Black et al. 2013).

Analysis and the minimization of the economic risks are part of the responsibility of the biobank towards the biobank donors, who entrust the biobank with their samples and data. Despite all efforts, there have been cases of public and private biobanks that needed to close due to various reasons, from failing to align interests with those of the stakeholders to competition for public funding at times of economic recession (Tupasela and Stephens 2013). Potential bankruptcy, loss of funding, closure of the hosting institution (Ciaburri, Napolitano, and Bravo 2016) may impact the biobank; there may be economic risks for the biobanks due to litigation that may result in the destruction of samples and/or a need to pay compensation (Caulfield and Murdoch 2017, Lewis 2015). In order to ensure continuation of the use or destruction of samples and data, there should be contingency plans, unless the actions to be taken are already determined by law. It should be noted, however, that good governance structures are central to ensuring public trust, maximizing contribution of biobanks to science and public health and minimizing the discussed economic risks.

#### Infrastructural: Technical risks

There are several technical issues related to risk management in biobanking. In most cases biobanks use freezers with a range of freezing temperatures, from –20 to –80 °C. There are also freezing solutions that can store samples at very low temperatures such as –150 °C and some samples (especially viable cells, cell lines) can also be stored in liquid nitrogen tanks either in liquid or in a vapor phase. The possibility of failures can be minimized; the biobank has to be ready for such possibilities regardless of the storage system used. The most common problem affecting the quality of samples stored in low temperatures relates to the electricity, i.e. power outage (depending on the local electricity infrastructure) or failure of the electric traction (unexpected repair time). In such situations, the biobank must have access to the electricity generator of the right capacity or an appropriate and large enough back-up system that will maintain the temperature in the freezers for a longer period (e.g., 24 h). It should be noted that the back-up system also has its limitations and requires servicing and care just like the equipment of everyday use. In the case of a back-up system that is based on cooling with CO_2_ and LN_2_ it is necessary to monitor the gas level on a regular basis, by weighing daily or checking gas level indicators. Gas supplies and cylinder replacement must be planned so as not to leave the main unit without a back-up system.

The risk of a mechanical failure can be minimized by keeping the equipment at the right temperature and humidity specified by the manufacturer and maintaining it regularly serviced; if it is already significantly worn out, a decision should be made to exchange it for a new one. Despite all efforts, the equipment might break down independently, and therefore it is imperative to create a back-up space where samples can be moved if the freezer or liquid nitrogen tank fails. To avoid sample loss, it is important to perform daily check-ups, both visually and audibly, to confirm that the equipment is working properly (including temperature monitoring). It is good practice to connect the temperature alarming system to the mobile phone of designated employees and to provide on-call service in case of failure. Nowadays, solutions such as Internet of Things allow remote and continuous monitoring of the storage conditions (e.g., via smartphone apps), allowing to check the equipment remotely, especially during holidays, weekends and non-working days. These systems must be protected from unauthorized control of the equipment. Recent hacking attempts of COVID-19 vaccine cold chain (Zaboeva and Frydrych 2020) suggest similar developments must be prevented in the biobanking sector. Finally, each biobank should have operational procedures in the event of an accident, the content of which is regularly validated.

Disasters that have affected biobanks are relatively rare and considering they are often unexpected, they may lead to the loss of a significant amount of collected biological material. In 2011, the loss of blood, urine samples and cell lines at the Danish Diet, Cancer and Health Biobank in Copenhagen made news when the biobank got flooded due to the heavy rainfall, during which the rainwater sewage system was unable to collect excess water (Roswall et al. 2013, Vogel n.d.). This case is often discussed in biobanking trainings as it demonstrates for the community that the location of the biobanking facility must be carefully planned and the lower ground floors or basements may be a serious threat to samples in the event of a flood. Global warming and changes in weather conditions may result also in other extreme weather events. For instance, in 2012, Hurricane Sandy hit the East Coast of the United States, severely affecting the overall infrastructure of biobanks in the New York area (Simeon-Dubach, Zaayenga, and Henderson 2013). Therefore, the location in premises that were originally intended for other activities requires a detailed analysis of the construction plan. The most important aspects are: building design to accommodate heavy-duty freezers and a backup system (checking the bearing capacity of the ceilings with a construction designer or architect), adequate and powerful electrical network to protect against fire due to short circuit caused by an overload in the electrical network, the ventilation and air-conditioning system that can adjust the air temperature and humidity, and finally, easy access for the transport of heavy goods such as gas cylinders, LN_2_ tanks and freezers (access to the freight elevator, wide entrance).

Biobanks collect and store a wide variety of samples, and these collections can be organized according to different protocols. The majority of biological sample collections for research projects are established on demand, where validated processes are generally applied to various stages of the specimen collection, as well as special operating procedures are created to ensure high sample quality and integrity. However, in some cases, biobanks also work with the biological material that is a residual tissue, for instance, from the diagnostic process. Such samples are burdened with the influence of other procedures, various environmental factors and may affect experimental results substantially. Considering that these samples may be the only ones that can be obtained due to their uniqueness, in such cases, material qualification or quality stratification procedures may be performed with these materials to exclude any potential biases. For instance, quality control assays performed in order to qualify clinical biospecimens can be applied to the specific disease area or particular downstream analytical platform. If such procedures are impossible, there are analytical assays that can be performed to stratify clinical samples according to their biomolecular quality (Schwarz et al. 2019). In this regard, while there may be differences in processes of collection, storage, quality control of primary and residual sample collections, as well as between clinical and research settings, there are various established procedures to limit the risks related to the biological integrity of the samples.

The problem of data mixing or wrong assignment of data to human biological samples is becoming less common but still occurs. Although most biobanks are now equipped with appropriate IT tools such as biobank information management systems (BIMS), some biobanks still store data in spreadsheet format or enter large amounts of data manually. The practices are often tied to economic factors and the level of investment in biobanking infrastructures. The other frequently encountered issue is the problem of data migration between old and new systems, especially if the migration is carried out by personnel who do not have the necessary expertise. The essential step is to validate the whole process after its completion. This is usually done on a random basis by selecting individual data or sometimes by specific collections being traced. The other issue related to data consistency, traceability, was described by Holub et al., who underlined the role of consistent and algorithmically harmonizable semantics of the information, so that the data search and exchange is possible via efficient search or filtering services (Holub et al. 2016). In 2012 Merino-Martinez described the work initiated by BBMRI.se on the Minimum Information About BIobank data Sharing (MIABIS) using semantic interoperability through harmonized services and common ontologies in order to minimize the lack of standards and generic solutions for interoperability and information harmonization in biobanks (Merino-Martinez et al. 2016).

Investments in the appropriate data security software should not become sidelined due to the fact that expensive equipment for processing and freezing biological material is prioritized. Biobanking data must be protected and this may be considered in various situations: for instance, deliberate attacks on the database in order to obtain sensitive information, burglary, software error or any other unexpected event (Sargsyan et al. 2020). Recent cybercriminal activity targeting health infrastructures have been identified in various countries, including the textbook cases of ransomware attacks on university hospitals in France in 2019 and Germany in 2020 as well as ransomware attacks on pharmaceutical companies, where cybercriminals may threaten to leak health data to the public or prevent further use of the IT infrastructure by encryption, unless a payment is made (Federal Office for Information Security (BSI) and Agence nationale de la sécurité des systèmes d’information (ANSSI) 2020). To prevent such attacks, research efforts towards proactive cyber and physical security at health infrastructures are expanding with large-scale international projects, such as SAFECARE (https://safecare-project.eu/), AI4HEALTHSEC (https://ai4healthsec.eu/) or HEIR (https://heir2020.eu/). Just as in the health infrastructures, protection against cybersecurity threats, such as hacking / data leakage, should be a key element of daily biobanking management. Secure data storage and analysis should be given the same importance as equipment failures and other disasters in biobank SWOT analysis, staff training and mitigation plans.

#### Infrastructural: Employees’ risks

Biobank activity includes management of risks involving employees, such as accidents due to various reasons (e.g., lack of training of staff and negligence). Certain biosamples are presenting specific hazardous properties which need to be addressed in the design of the biobank in order to avoid or mitigate risks of contaminations for biobank employees and broader environments. Protection of the employees is an ethical duty and in Europe, for instance, the European Union (EU) Directive 2000/54/EC (European Parliament and Council of the European Union 2000) fixes specific rules for protecting workers exposed to infectious biological agents (micro-organisms, cell cultures and human endoparasites), as a specification of the general Framework Directive 89/391/EEC (Council of the European Union 1989) on health and safety at work which applies to any sectors of activity, including research biobanking.

The Framework Directive includes an obligation for the employer to ensure the safety and health of workers in every aspect related to work, without imposing financial costs to the workers to achieve this aim. The general risk prevention approach includes the following principles to be embraced by the employer of the biobank staff. First, the employer must evaluate the risks for the employees. Second, employers must plan specific measures for combating the risks at their source, meaning at the time of sampling where the biobank is legally integrated in a health establishment, or at the time of the sample entry where the biobank operates as a separate legal entity. Third, where a risk remains, the employer must secure the facilities for the storage and handling of dangerous biological samples and provide relevant equipment for the employees.

In biobanking, it is important to ensure that the entire circuit for the biological samples processing conforms with high quality and security standards and that these standards are adequate for receiving biosamples. Securing the workers’ activities requires continuous attention and includes specific work to prioritize collective protective measures for employees. Where necessary, the employer must take steps to adapt the work and workplace to individual particularities (e.g., employee’s disability or specifically exposing task). According to the Framework Directive, the employer must in particular consider the worker’s capabilities with regards to health and safety in entrusting tasks to workers. The employer must inform, train and consult workers, in particular on introduction of new technology, and allow them to take part in discussions on all questions relating to safety and health at work. This includes individual training ensuring that each worker receives adequate safety and health protection capacity. The employer must designate worker(s) to carry out activities related to the protection and prevention of occupational risks and take the necessary measures for first aid, firefighting, evacuation of workers and action required in the event of serious and imminent danger. A list of occupational accidents must be kept, and occupational accidents suffered by the workers must be reported to the responsible authorities.

Employees have also duties with regard to security and safety at work which are usually contractual (employment contract). The worker must specifically respect the instructions given by the employer regarding the occupation. This includes a duty for the employee to make correct use of any equipment, other means of production and personal protective equipment at disposal, to immediately inform the employer of any work situation presenting a serious and immediate danger and of any shortcomings in the protection arrangements, as well as to cooperate with the employer in fulfilling any requirements imposed for the protection of health and safety to ensure that the working environment and working conditions are safe and pose no risks. However, even when the employer subcontracts certain services regarding security and safety at work, the employer is still fully responsible of the choice of the subcontractors and for consistent and efficient security and safety at work, including regular updates and investments in order to ensure its continuous appropriateness and resilience.

Risks related to the hazardous characteristic of a sample are central to employees’ safety and a classification is fixed by law according to the specific risk of the biological agent (European Parliament and Council of the European Union 2000). Groups of biological agents range from no-risks (Group 1), including most common categories of clinical pathological samples, to high-risk agents (Group 4), where latter may cause severe human disease and present a high risk of spreading to the community. For those high-risk materials there is usually no effective prophylaxis or treatment available. The containment of such risky samples, the equipment of employees, the related access and transport procedures (e.g. package characteristics and labelling) must be tailored to these risks. Sample collection during the pandemic made evident both the risk of infection and the necessity for precautions; e.g., biosafety level 2 for non-propagative diagnostic laboratory works with SARS-CoV-2 and biosafety level 3 for other works such as viral isolation or for handling live culture of the virus (Hofman et al. 2020).

While the first objective of these rules is to protect biobank employees’ safety and health, such measures also indirectly allow preserving samples from external contamination. Occupational safety and health protection issues must also be considered during the access requests to the biobank materials: first, for allowing the proper preparation of the samples before any external release and transportation, and second, to ensure appropriateness with regard to the user needs, capacities and guarantees. For instance, traceability becomes one of the key concerns for samples collected during the COVID-19 pandemic since contaminated samples, e.g. cancer tissues, may pass on viruses such as the SARS-CoV-2 to the recipients (Hofman et al. 2020). Where feasible with regard to the use purpose claimed by the applicant for access, the biobank management team should seek replacement of the dangerous samples by non- or less dangerous materials. In any case, the recipients must be informed about the risks related to the handling of the samples accessed and must, in return, ensure that they have the relevant expertise and facilities. A competent transportation service which will comply with the regulations on the transport of dangerous goods may be necessary. All these aspects, including insurance and liabilities, are usually specified by the contractual clauses of the material transfer agreement (MTA) between the biobank and the recipient(s).

Significant accidents must be reported to competent national authorities. Neglecting employees’ security can lead to life-threatening events and eventually to a broader risk, threat or damage to environmental and public health. Furthermore, there is a risk of damaging the reputation of the biobank and its host institution. It is thus particularly important to refrain from opening a sample storage/processing service in a rush, including for financial reasons, and to put in place all the necessary safeguards before involving employees.

#### Participation and activity risks

Biobanks have to prevent certain risks for their operation and sustainability by increasing their capacities and competences to engage potential participants and/or build trust. These risks include low participation, a substantial number of withdrawals and recruitment bias. Successful operation of biobanks depends on the willingness of potential donors to take part and share their samples and data. Results of comparative studies show that public trust and support for biobanks and biobank-based research in European countries is variable, e.g. potential donors in southern and eastern Europe show lower willingness to participate in comparison to north-western countries (Gaskell et al. 2013). In the context of genomic research there are also differences between European countries and globally in levels of public trust in different actors (medical doctors, non-profit and commercial researchers, governments, etc.). For example, the United Kingdom (UK) population shows higher levels of trust than the populations of Germany and Poland (Middleton et al. 2020). These studies also show that trust and willingness to participate depend on a range of factors, including people’s engagement with biobanks, concerns about privacy and data security, trust in the socio-political system, key actors and institutions involved in biobanking (Gaskell et al. 2013). In different parts of the world reasons for lack of trust may vary. For instance in South Africa, research suggests power asymmetries and a general distrust in science based on experience of historical exploitation as important factors, which might be ameliorated by building robust independent governance structures (Moodley and Singh 2016). Studies also show that potential donors are less willing to donate samples and data to for-profit users than they are to non-profit institutions and researchers; the main concerns of members of the public in the context of commercial biobanks relate to benefit sharing, profit making and control over samples and data (Middleton et al. 2020, Nicol et al. 2016, Broekstra et al. 2020). There are suggestions to reduce prejudice against commercialization with good, independent governance of biobank resources and transparency regarding commercial involvement (Nicol et al. 2016).

Loss of trust might also be a reason why donors withdraw their samples and data from a biobank. The right to withdraw is a fundamental right of research participants and biobank donors explained in the process of acquiring informed consent. Participants can withdraw their consent at any moment without further explanation and negative consequence. At the same time the specific structure of a biobank and design of biobank-based research studies may put certain limitations on withdrawal, which must be explained to the donor (Melham et al. 2014). Distinct ethical problems are relevant regarding withdrawal of samples and data in the case of minors. Since minors are not capable of consenting at the time of donation, there is a consensus that parental consent has a temporal scope and minors should be re-contacted after reaching maturity to be informed about their rights, e.g., to withdraw samples and data from a biobank (Hens et al. 2011, Council for International Organizations of Medical Sciences 2016). This process should be well-planned to reduce the risk of a high number of withdrawals.

Another risk to be prevented is recruitment bias. Some research studies show that recruitment of donors for biobanks tends to prioritize certain groups – white, middle-class, more highly-educated, females – and to exclude or underrepresent other groups (Prictor, Teare, and Kaye 2018, Haddow 2009). For example, a study on representativeness of the UK Biobank cohort showed that the participants were more likely “to be older, to be female, and to live in less socioeconomically deprived areas than nonparticipants”, as well as “less likely to be obese, to smoke, and to drink alcohol on a daily basis and had fewer self-reported health conditions” than the general population (Fry et al. 2017). Recruitment biases are also reported regarding other biobanks and biobank-based research studies (Leitsalu et al. 2015, Bisgaard et al. 2013, Haddow 2009). Age, place of residence, cultural sensitivities, digital gap and issues of literacy and language are mentioned among barriers to participation in biobanks (Prictor, Teare, and Kaye 2018). ‘Volunteer effect’ is another reason for recruitment bias; those who decide to volunteer may differ in some important traits compared with those who do not volunteer (Fry et al. 2017, Bradburn et al. 2020). Recruitment bias infringes on the principle of justice, influences representativity of biobank collections and has implications for the generalizability of research results and ability to reach full statistical power. Some authors suggest that tools for overcoming recruitment bias include dynamic consent (Prictor, Teare, and Kaye 2018) or research on reasons for non-participation (Haddow 2009).

While there are risks related to participation and activity, it must be noted that biobanks have been expanding in numerous countries in recent years, leading to increased awareness and best practices. As with other risks mentioned in this section, to minimize risk of low participation, it is important to increase trustworthiness and context-specific trust, to familiarize the public with the purposes of biobank-based research, develop exemplary models of conduct, implement transparency, build independent governance structures, maximize societal benefits resulting from the research and authentically engage with the public (Gaskell et al. 2013, Middleton et al. 2020, Moodley and Singh 2016, Ursin et al. 2020).

#### Research community risks

Biobanks are both part of and provide service for the research community. Considering this dual function, they are affected not only by their own activity, but also the research activities of the institutions and individual scientists who make use of the samples and data that they hold. This means regardless of the ethics evaluations, stringent access criteria, Material Transfer Agreement/Data Transfer Agreement (MTA/DTA) clauses, the use of samples and data by the researchers always involves certain risks. These include risks that we have already mentioned above, such as risks for the employees or technical risks such as data security (e.g., hacking) or contamination, but also intentional or unintentional misconduct, such as use of samples and data for purposes not agreeable to the conditions set by the biobank or the participant or release of data to open access databases that may violate the participant’s privacy.

Scientific misconduct or inappropriate use of samples and/or data in one context may result in important consequences for the biobank, not only as an individual institution that was involved as an intermediary between the participant and the research institution, but also for the entire biobanking and research community. In other words, the research community risks here are twofold: on the one hand, a controversy at one biobank has the potential to influence the public trust in the other biobanks; on the other hand, any negative publicity may in a worst-case scenario destabilize the research communities’ and medical establishments’ cooperation and collaboration with a specific biobank or biobanks in general. A major example in this regard is the Havasupai controversy, as mentioned above. Members of the Havasupai, a native American tribe near the Grand Canyon, were recruited by Arizona State University scientists for diabetes research due to high incidence of type II diabetes in the community, but the samples and data were also used for researching schizophrenia and migration (Garrison 2012, Quinn et al. 2013, Tzortzatou 2015, Harmon 2010b). Upon learning about the other research projects, participants sued the researchers and the court ordered the return of the samples, payment of direct compensation at an order of 700,000 USD and further compensation as infrastructure, while the researchers got a banishment order, meaning they were not allowed to enter the tribal territory (Garrison 2012, Harmon 2010a). On top of this court order numerous native American tribes and organizations reacted, some of them with moratoria on genetics research (Garrison 2012). Such distrust among different populations towards the research community as a result of the actions of few researchers may carry risks for representativeness and usefulness of findings as well as concerns for participation bias and societal justice. Similarly, genomics research on same-sex sexual behavior (Ganna, Verweij, Nivard, Maier, Wedow, Busch, Abdellaoui, Guo, Sathirapongsasuti, Lichtenstein, et al. 2019), conducted with data from UK Biobank, opened up questions into whether this falls into an acceptable research category considering that the biobank’s participants have agreed to only health-related research (Goisauf, Akyüz, and Martin 2020, Holm and Ploug 2019, Ganna, Verweij, Nivard, Maier, Wedow, Busch, Abdellaoui, Guo, Sathirapongsasuti, Team, et al. 2019). In this case, whether participants would have agreed to the decision of the biobank to consider genetics of sexual orientation under the ‘health-related’ research category and the consequences of this decision are unclear.

It is worth noting that policy and regulatory frameworks are often political and certainly evolve over time. Such changes may bear potential challenges since the institutional, regional, national or international policy and regulatory frameworks may not be completely aligned. Above challenges may be addressed in multiple ways, from lobbying for laws to educating the biobank staff and decision-makers in research ethics, to building dynamic and resilient governance mechanisms with transparency and continuous engagements with the stakeholders. Due to the scope of the paper, we do not explore this topic further; however, we note that this is a potential topic for future research.

Biobanks, like other academic units conducting research, may also run into funding problems at mass scale in case the public mistrust, controversies, or lack of initiative for collaboration supersede the expected benefits from these infrastructures. For instance, it is possible that a mismatch between the ‘projected’ and ‘produced’ usefulness of the material and data, e.g. in federated systems or infrastructures bringing together smaller biobanks, may hinder realization or continuation of such efforts (Aarden 2017). Considering that funding choices are themselves political decisions and are embedded in imaginaries of certain futures often at national and international levels, lowering of funding at local or international scale may hinder the research community’s capacity to share and access samples and data. Not only ensuring good governance at a biobank, but also striving towards similar standards across the biobanking and research community are key to minimizing risks at institutional and community levels.

### Participant’s risks

The second perspective used for the construction of the typology is that of the participant. Four major types of risks have been identified as salient: physical risks that directly involve the participants, informational and/or privacy risks, risks related to findings that are often considered under incidental/secondary findings, and finally the values at risk.

#### Physical risks

Although collection of biological samples for biobanks usually poses minimal or minor risk, specific physical risks may exist for the sample donor, depending on the procedure used and the context in which the samples are obtained. The physical risks for the participants are mainly present at the very beginning of the biological sample collection activity, but specific ethical concerns emerge throughout the process. Communication of risks and associated measures to protect donors should be included in the informed consent process, as specified by rules for obtaining lawful and ethical consent to medical interventions, including experimental ones: the EU law, i.e. the Clinical Trial Regulation/CTR (European Parliament and Council of the European Union 2014) and the General Data Protection Regulation/GDPR (European Parliament and Council of the European Union 2016), national laws and relevant ethical recommendations together with internationally recognized Good Clinical Practices (European Medicines Agency 2016, The Commission of the European Communities 9.4. 2005). Biobanking samples and data for research should necessarily be considered and clearly indicated to the potential donor in a responsible and transparent way, prior to the sample collection or as soon as possible if the donor was unable to provide consent at the time of the intervention. Policy documents and results of research studies emphasize not only the importance of the content of written consent form, but also the interactive discussion, explanation of risks and benefits in a simple, clear, and meaningful, dialogical manner, as well as answering questions and assessing the level of understanding of the patient regarding the information provided, as crucial parts of the informed consent process (Council for International Organizations of Medical Sciences 2016, Nusbaum et al. 2017, Xu et al. 2020). Besides the risks of the procedure, the individual should be informed about the biobanking purpose(s) and have the possibility to refuse participation or request additional details at any time. This is the first building block of the ethical basis of the biobank activity. It is also the starting point of the traceability of the samples and associated data.

Sampling can be performed by using invasive or non-invasive procedures which can either be part of the patient treatment (e.g., diagnostic procedure, surgery) or could be supplementary procedures, including experimental or innovative and yet unproven procedures performed for scientific research and technological development purposes (e.g., through the use of innovative encapsulation device on humans). While there is no universal definition of invasiveness regarding a medical act (Cousins, Blencowe, and Blazeby 2019), sample collection can imply the use of invasive medical devices which will be used to gather the samples. The EU Medical Device Regulation defines “invasive device” as “any device which, in whole or in part, penetrates inside the body, either through a body orifice or through the surface of the body” (European Parliament and Council of the European Union 2017). Therefore, all the sample collection techniques necessitating the alteration of the human organism, crossing the cutaneous/mucous barrier, such as the collection of blood samples, of bone-marrow, through punctures, or the collection of cancerous samples during surgical acts are considered invasive. Invasiveness could also include activities which do not cross the cutaneous barrier, but which include such a potential risk, for example through the use of an experimental self-administrated sample collection device for human papillomavirus (HPV) testing. Other techniques could qualify as invasive, where they entail a risk of dissemination of a product or a substance into the body, or a risk of impacting healthy tissues or organs.

The risks can be multiplied for the sample donor if an invasive procedure is repeated for the need of a specific research project or for biobanking purposes, particularly where the donor is a healthy volunteer and where the procurement act is not performed under appropriate medical supervision. The notion of invasiveness is also subject to cultural appreciation and could be interpreted differently. Depending on the context and nature of the intervention, the risk scale can range from low to high risk, and necessary measures to mitigate these risks and other potential discomforts for the participants must be considered and implemented before the collection of any samples. In this respect, the appropriateness of the qualifications of the staff involved in the sample collection and of the collection site (e.g., hospital’s surgical department), as well as quality of informed consent are particularly important. Insurance for research participants could be mandatory.

Sampling can also consist of non-invasive procedures which do not involve physical risks for the donor as defined above. Such procedures include, for example, collection of bodily materials or substances which are naturally secreted or rejected by the human body, such as saliva, stool, or urine. Collection of samples from deceased persons’ remains should fall under this category, as well as other activities which do not qualify as invasive in specific legal or cultural contexts.

In any case, a sample collection process should follow a detailed protocol, which should fix adequate number of specimens, quantity or volume of the samples with regard to the analytical purpose and quality standards to be complied with. In order to lower the risk of repeating the procedure, the procedure must ensure rapid stabilization of the samples and first storage conditions as well as proper annotation, coding, packaging and labelling of the sample. Sample and personal data collection processes must follow a minimization approach in order to ensure both sufficient quality of the sample for research uses and minimized physical and privacy risks for the donor.

#### Informational/Privacy risks

Another risk area is associated with the processing of data, be it in regard to fraud, unintended use or re-identification of data subjects. Data breach is one of the most discussed risks from the participants’ perspective regarding the processing of their personal data in biobanks, especially in the case of genetic data (Hautala n.d.). Furthermore, there are concerns that government, insurance companies or employers might misuse genomic data to discriminate against persons (Milne et al. 2019).

Informational and privacy risks are mainly codified in the legal realm and the discussion of risks often relate to the genomic data. In the European case, GDPR’s “risk management approach” both by design and by default is the complete anonymization or pseudonymization of genetic data by using state-of-the-art technical means and safeguards (Tzortzatou et al. 2021). In this risk management approach, data protection impact assessment (DPIA) plays an important role; however, the risk of re-identification is often difficult to assess. For instance, genomic data, which could be considered anonymized at one stage, may be rendered personal data at a later stage, due to the numerous connections among genetic datasets (Harbord 2019), increasing the possibility of re-identification even in those cases where the individuals have not provided their own DNA to a database (Shabani and Borry 2018, Borry et al. 2018). Nevertheless, some scholars argue the importance of the complete opposite – being able “to be re-identified” as an individual/patient – in case the individual could benefit from potential future research results, e.g. the case of rare diseases (Hansson et al. 2016). Thus, efforts to counter the risk of re-identifiability may contribute to the risk of inability to benefit from research results.

Finding the balance between above-mentioned privacy risks and public benefit from scientific research appears even more challenging in light of the rapid development of technologies. In such a setting, fear of an increased risk of genetic discrimination is more present than ever. National laws such as the Health Insurance Portability and Accountability Act/HIPAA (US Department of Health & Human Services 2012), the Genetic Information Nondiscrimination Act/GINA (United States. Congress. House. Committee on Energy and Commerce. Subcommittee on Health. 2008), soft law guidelines such as the United Nations Educational, Scientific and Cultural Organization (UNESCO) Universal Declaration on the Human Genome and Human Rights (UNESCO 1998) and binding international legal instruments explicitly refer to the prohibition “of genetic discrimination based on genetic characteristics” (Godard et al. 2003, Gammon and Neklason 2015, European Parliament, Council of the European Union, and European Commission 2012). Concerns regarding the use of genetic information by insurers and employers vary among several scenarios such as the risk of rating up insurance costs, the risk of getting denied insurance due to genetic testing results of individuals or their close relatives, the risk of getting fired or not being recruited, and the risk of being socially excluded among others.

Methodological and technological developments in data sharing and use, the amount of data collected globally, and new methods of analysis have resulted in a change of the risk profile, particularly with regard to the increasing scale of the use of health data and related challenges for informed consent models in biobank-related research (Mikkelsen et al. 2019). An important aspect of this change is the temporality, where risks related to the future use and sharing of data have raised concerns about potential harms related to the protection of privacy. This is particularly important in connection to genetic analysis and the potential risk of re-identifiability, discrimination, but also to stigmatization and participants’ concerns about biobanking (Helgesson 2012, Kasperbauer et al. 2018). Even when discrimination or social exclusion is not evident, the possibility of it may produce stigma at the individual level. The individual may believe that others are aware of the stigmatic condition (discredited stigma) or that stigma is not immediately evident to others (discreditable stigma), for example as in the case of breast and ovarian cancer risk due to *BRCA1*/*BRCA2* mutations (DiMillo et al. 2013). Considering the temporality, the risks associated with the personal genomic data at biobanks and possibility of discrimination at the current moment cannot exclude worries about stigmatization in the future with developments in the data and technical infrastructures in sight.

Finally, studies have shown that information about data uses is vital to approach concerns about data protection and possible uses beyond the original research context which influence participants’ opinions towards consent models and biobank research more generally (Goisauf and Durnová 2018, Joly et al. 2015, Petersen et al. 2014). Correspondingly, survey results representing the perspective of biobank professionals indicate that informing participants about data sharing and multiple uses of data in informed consent procedures need to be improved, together with participant engagement (Goisauf et al. 2019). In conclusion, there is room for improvement in managing informational risks, including the way research participants should be engaged with and informed.

#### Risks related to individual findings

Incidental finding is a research finding “concerning an individual research participant that has potential health or reproductive importance and is discovered in the course of conducting research but is beyond the aims of the study” (Wolf et al. 2008). In general, incidental findings are conceptualized more as a benefit and less as a risk, especially in case of medically actionable findings. In case the research participant would not like to know or would like to exercise her right not to know according to the Oviedo convention (Council of Europe 1997, 2008), incidental findings might pose a risk of moral dilemma, especially if researchers feel a strong duty to inform the donor. The risk of moral dilemma, however, may also be high on the participant’s side as the participant may feel the obligation to inform biological relatives of the findings, which may have consequences for them. Risk is also linked to cases of false-positive or false-negative incidental findings or reporting on non-significant findings that may result in unnecessary anxiety or excessive clinical assessment (Gibson et al. 2018). Currently, the data on positive or negative consequences of returning incidental findings in the context of biobank-based research is limited, and it is often difficult to determine whether a particular incidental finding may result in a clinical benefit (Gibson et al. 2018). Research studies show that in order to diminish risks in management of incidental findings, it is important to clearly assign the roles and responsibilities and develop detailed policies (Wolf et al. 2012).

A large group of incidental findings are generated by genomic research and specifically by whole genome sequencing. Globally there are different guidelines regulating management of incidental findings resulting from genomic research. For example, in 2013 the American College of Medical Genetics and Genomics (ACMG) introduced recommendations for reporting incidental findings in clinical exome and genome sequencing, including a list of 56 genes for 24 inherited disorders (Green et al. 2013). ACMG guidelines have been broadly used and referenced, but at the same time also criticized for demonstrating an overly paternalistic attitude and taking into account only professional standards, not individuals’ interests and preferences (Ploug and Holm 2017). The ACMG guidelines were amended in 2016 to allow opt-out similar to ‘the right not to know’ (Kalia et al. 2017). A different approach was suggested by the European Society of Human Genetics, mainly stating that unsolicited genetic variants should be returned only if the finding is informative about a serious health problem for the donor or her relatives (van El et al. 2013). Analysis of guidelines shows that various approaches to returning incidental findings implemented globally may be contradictory and harmonization of requirements is necessary (Thorogood, Dalpé, and Knoppers 2019).

Other types of biobank-based research providing incidental and secondary findings, as well as individual research results also raise discussions on ethical challenges. One example is microbiome research providing information on susceptibility to certain diseases which potentially may lead to risk of hype and targeted advertising of different products to those who are informed as being at higher risk of particular diseases or conditions (Ma et al. 2017). Some incidental findings have potential reproductive importance and may affect reproductive decision-making by providing information on health risks for (potential) descendants. For these findings it is especially important to ensure that information is provided by adequately trained professionals in a manageable and comprehensible way, research data is translated into meaningful information, and donor’s expectations and worries are addressed (Horn and Parker 2018).

#### Protection of values: Values at risk?

Biobanking is based on the value of solidarity due to its constitution of a long-term resource for humanity as a not-for-profit biological legacy of past and present generations, which shall serve public interest in health, noting that for-profit biobanks also exist. Present sample donations always represent a potential for the future of health, intergenerationally, interculturally, internationally, as well as for technoscientific developments that cannot be foreseen at present. The values of inclusivity and sharing at the center of research biobanking aim to serve the collective scientific use of the samples in order to develop biomedical knowledge which could ultimately serve individuals around the world with all their differences, emphasized with the imaginary of precision medicine. Giving samples for health research is often framed as an altruistic humanitarian act contributing to present and future human health while data sharing has recently been framed as data altruism (Kibbe 2016). In this perspective, biobanks are responsible for ensuring the conditions of trustworthy management of the samples, minimizing risks of misuses of the stored samples while maximizing their legitimate uses in research. Since participants provide their samples and data for advancing scientific research for valid purposes, the inaccessibility of or inability to use the samples stored create ethical tensions between the initial wish of the donor to actively contribute to science and the potential restrictions which could result from a biobank’s access policies. Ensuring adequacy between the desired sharing of the samples based on individual consent and other interests a biobank could have in impeding access to the same samples should always be an ethical concern for biobank access management bodies, such as data access committees (DACs).

Biobanks are largely dependent on public support and the donations of biomaterials and data from patients and research participants according to their informed consent. Losing the support of these stakeholders constitutes a major risk for biobanks’ sustainability. Participation in biobanks is associated with certain expectations and values, such as altruism and solidarity that motivate potential participants to contribute to health research and thus to the good of the society. Cases in the past have pointed to serious consequences that can arise from violating the values of participants and other stakeholders. The risks regarding values often result from a discrepancy between the imagination about value generation and the ultimate use of the donated samples and data.

Hence, biobanks can take an active part in the construction of values that they themselves could risk violating (Reardon 2017). Several initiatives have been established that aimed to source biovalue (Waldby and Mitchell 2006) from the genomes of a nation’s population, for example deCODE Iceland, UK Biobank, the Estonian Genome Project and Generation Scotland. Based on the desire to generate knowledge about the genomes of the nation, these were seen as a resource that would in turn benefit the population, and would sooner or later become an investment in an international bioeconomy (Reardon 2017). This often resulted in a break with the values of the participants that their donation would primarily contribute to the good of the nation, for example in the form of knowledge generated within the country (Reardon 2017).

Previous research has shown that citizens and biobank participants have an image of an “appropriate” use of biomaterials and data in research, which often excludes use for profit making or use outside health research (Goisauf and Durnová 2018). Such circumstances became apparent, for example, in the case of insolvency of a Sardinian biobank and related questions about the transfer of collected biological samples and genetic data to another data controller and the rights of donors regarding consent (Piciocchi et al. 2018). Despite established systems of control, some research may be considered to be in the gray zone. For instance, discussion is needed regarding access to data for health-related research and what ‘health-related’ in broad consent does or does not include (Holm and Ploug 2019). Similarly, as the Havasupai case has shown, certain research that the participants do not approve of, such as association of a stigmatizing disorder with the community or scientific conclusions that go against the foundational beliefs of the community may create tensions between researchers and the participants, as far as to cause the complete collapse of the communication (Harmon 2010a, Garrison 2012). In this regard, biobanks’ engagement practices can be instrumental in understanding and upholding values that are central to the participants and other stakeholders.

## Discussion

From conceptualization to developing into a well-functioning institution, biobanks have to consider risks from multiple perspectives for successful continuation of their activity and protection of participants as is true for other research units. Preparation of standard operating procedures, training of staff, updating IT and physical infrastructures contribute to minimizing risks. However, regular risk assessments according to current standards capture only a fraction of risks at a biobank that has a specific infrastructural hinterland (i.e. institutional history, research and academic involvements, expertise) and is embedded in a distinct societal context. With this typology of risks, we are providing a collection of risks that may conceptually apply differently to individual biobanks; however, in developing this typology, we have shown that there is an entanglement of risks and we argue that an adaptive risk governance is mandatory for success of a biobank.

The numerous cases mentioned so far are perfect examples to discuss how in fact risks are interrelated and thus necessitate a holistic thinking. Risk is often discussed through the focus on participant’s risks in the biobanking and ethical, legal, societal implications (ELSI) literature. With the risk typology, we have discussed risks that directly relate to the biobank as an institution or the individual as a participant, but also indirectly to the success of the biobank and the research community or societal support for biobanking. We have highlighted that risks cannot only be thought of as material or physical, but may be embedded in values, practices, relationships, as well as broader phenomena, such as research integrity, representativity and societal justice.

**Fig. 2:**
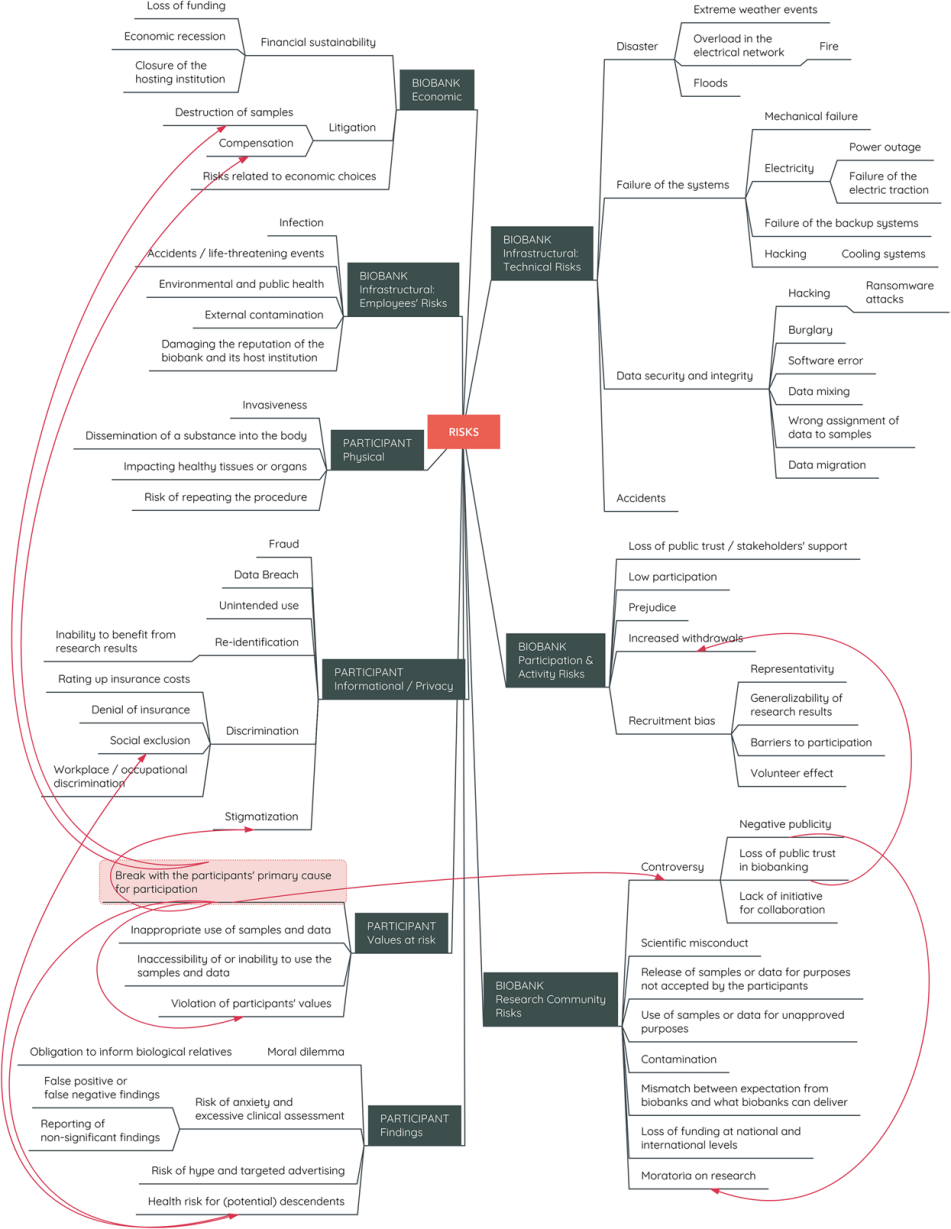
The entanglement and multiplicity of risks in biobanking. The red lines indicate the entanglement of the realized risks within the Havasupai case, starting with ‘break with the participant’s primary cause for participation’

The entanglement of the risks is evident in two ways: first, certain risks may be contributors for other risks (downstream/upstream risks), and secondly, a risk may exhibit multiplicity. For example, the Havasupai case highlights the potential problems that may be caused by not communicating the scope of biomedical research sufficiently (Fig. 2). Seen as a legal document, a signed informed consent form may give the impression that the participant has knowingly agreed to the conditions; however, verbal communication in this case has misled the individuals as a community (Havasupai tribe) towards thinking that their samples will only be used be for diabetes research, whereas the conducted research included schizophrenia and genetic ancestry/migration (Harmon 2010a, Garrison 2012).

This controversy shows that many components of the typology are highlighted together, some of which are consequences of the others. The risk of the break with the participant’s primary cause for participation is realized when along with diabetes, other research purposes emerged for the samples. Furthermore, the risk of “violation of participants’ values” materialized because for the Havasupai tribe, their disputed (genetic) origin in the Grand Canyon, is also opening to debate the tribe’s foundational myth and, possibly, the basis for the tribe’s claim to the territory (Harmon 2010a). Use of the samples for schizophrenia research (Garrison 2012), on the other hand, highlights the informational risks for the participants as it carries both a stigmatization and discrimination risk, where belonging to the Havasupai population may cause social exclusion or occupational discrimination. Similar concerns can be raised for health risk of potential descendants or non-participating members of the community, who did not contribute to the research with their samples but are affected by the findings.

For the biomedical research community, the Havasupai case became a full-blown controversy appearing in the media and academic publications, resulting in negative publicity for the hosting institution, Arizona State University, and may have also resulted in loss of public trust in biobanking or biomedical research within and beyond the Havasupai community (Garrison 2012). While the Havasupai community sued the researchers and two further risks – risks of compensation and destruction of samples – materialized as a result of the litigation, for the research community, another risk emerged as many other tribes claimed moratoria on genetic research, negatively affecting public opinion in different communities towards contributing their samples and data to different life sciences research projects (Tauali`i et al. 2014, Garrison 2012). Such developments result in recruitment bias and, as downstream risks, may cause representativity problems and lower generalizability of research results.

Biobanks by definition include numerous actors both as part of a broader ecosystem that comprises various stakeholders, but also within an institutional setting that may have extensive compartmentalization and expertise, from the role of the quality manager to that of the genetic counsellor. Risks that may exist at different levels may not be self-evident at other levels, especially if the governance structure does not allow productive exchanges between different components of the biobank. For instance, the risk of deliberate attacks, such as hacking, are included under various categories in the typology. Under the technical (infrastructural) risks, hacking is considered as a risk not only in the obvious data security and integrity type. The same risk is also included under failure of the systems with the example that online control of the cooling system from outside the biobank poses risk of loss of temperature control as a result of hacking of such technologies. Although this has not been reported to our knowledge, similar developments were reported for the safekeeping of the COVID-19 vaccines as stated above (Zaboeva and Frydrych 2020). Similarly, hacking can be counted under informational/privacy risks of the participant, both due to potential leak of data from the biobank, as cybersecurity incidents at public hospitals and pharmaceutical companies have shown (Federal Office for Information Security (BSI) and Agence nationale de la sécurité des systèmes d’information (ANSSI) 2020), but also as a result of the participant’s actions as the web-based dynamic consent models will possibly be more widespread in the future. Hacking attacks are only one example of multiplicity and it highlights the importance of continuous updating of the risk assessment as well as engagements between different stakeholders and among biobank staff.

The typology provided here as an end-product is possibly less useful for a biobank’s risk assessment than the exercise of producing such a typology. With this typology, we stress the importance of an adaptive risk governance and the identification of key actors over a simple risk assessment, as has been suggested in the literature before (Jacobson, McHugh, and Tran 2013). Based on the typology, such a governance model must be able to adapt to the technoscientific and infrastructural developments, but also be sensitized to societal and legal changes. While adapting to the present may be useful to counter foreseeable risks, there is also a necessity to imagine potential risks in the short- and long-term future (Gille, Vayena, and Blasimme 2020). Continuous engagements both within and beyond the biobank are crucial in sustaining critical assessment of the risks that can adapt to developments.

## Conclusions

Biobanks as research infrastructures are essential components of science supporting evidence-based developments in biomedical research. They have been expanding both in number and scale, and as part of broader networks. At the same time, there have been many relevant developments in different parts of the ecosystem in which biobanks are embedded, from the amount of data that are available and data analysis tools that are in the making, to changes in the regulatory environment. For such a rapidly changing and expanding sector, we have identified how risks in biobanking can be categorized as part of a typology that could help to improve existing risk management practices or design new exercises and approaches by proposing an adaptive risk governance. We have also claimed that such an exercise would best suit the specific context of a biobank if the typology is continuously updated according to the internal and external developments in ways that would allow exchanges between different stakeholders and elements of the biobank. The life sciences research community has immense experience with managing risks and this long-term experience may have been instrumental for the many success stories of biobanking. Biobanks’ systemic approach towards risks may benefit individual researchers and their research projects by raising standards also among the users of the samples and the data. Thus, while we acknowledge that current risk assessment and mitigation practices in the biobanking community are useful, we have concluded by proposing adaptive risk governance that would further strengthen and build on the adaptive capacity and knowledge of biobanks in this regard.

## Data Availability

Not applicable.

## Abbreviations

ACMG: American College of Medical Genetics and Genomics
BIMS: Biobank Information Management System
COVID-19: Coronavirus Disease 2019
CTR: Clinical Trial Regulation
DAC: Data Access Committee
DPIA: Data Protection Impact Assessment
DTA: Data Transfer Agreement
ELSI: Ethical, Legal, Societal Implications
EU: European Union
GDPR: General Data Protection Regulation
GINA: Genetic Information Nondiscrimination Act
GWAS: Genome-Wide Association Studies
HIPAA: Health Insurance Portability and Accountability Act
HPV: Human papillomavirus
ISO: International Organization for Standardization
MIABIS: Minimum Information About BIobank data Sharing
MTA: Material Transfer Agreement
SNP: Single Nucleotide Polymorphisms
UK: United Kingdom
UNESCO: United Nations Educational, Scientific and Cultural Organization
